# Are we socially equally at risk of smoking during the COVID-19 pandemic? French data from 2009-2021: Astrid Juhl Andersen

**DOI:** 10.1093/eurpub/ckac131.080

**Published:** 2022-10-25

**Authors:** AJ Andersen, M Melchior, M Mary-Krause, S Wallez, I Hecker

**Affiliations:** ERES, IPLESP, Sorbonne University, INSERM, Paris, France

## Abstract

**Background:**

Since 2020, the COVID-19 pandemic has greatly affected people including a significant increase in mental health difficulties. Cigarette smoking is found to be strongly associated with mental health conditions, which is why the pandemic might have influenced the secular decline in smoking rates observed over recent years. Persons belonging to socioeconomically disadvantaged groups may be particularly affected, both because the pandemic is found to exacerbate existing social inequalities and because this group was more likely to smoke before the pandemic. We examined the prevalence of smoking in a French cohort study, focusing on differences between educational attainment. In addition, we examined the association between educational level and interpersonal changes in tobacco consumption from 2018 to 2021.

**Methods:**

The TEMPO cohort study included 1785 French adults followed between 1991 and 2021. With four assessments of smoking status available before and two after the onset of COVID-19, we estimated the smoking prevalence over time stratified by highest obtained diploma. We studied interpersonal change in smoking status between 2018 and 2021 among 148 smokers, using multinomial logistic regression.

**Results:**

The prevalence of smokers was higher among those with low educational attainment compared with those with higher diploma at all timepoints. The difference between the two groups increased from 2020 to 2021 (4.8% to 9.4%). Smokers with high educational level were more likely to decrease their tobacco consumption from 2018-2021 compared to low educated smokers (aOR=2.72 [1.26;5.89]).

**Conclusions:**

Current findings showed a widening of the socioeconomic gap over time in smoking rates, which emphasizes the vulnerability of persons with low educational attainment to smoking, also during the pandemic.

**Key messages:**

• The existing gap in smoking prevalence between lower and higher diploma groups has increased from 2020 to 2021, which may be a consequence of the COVID-19 pandemic.

• From 2018 to 2021, people with high school as highest qualification were less likely to decrease their tobacco use compared to higher educated people.

